# Impact of trophectoderm biopsy on serum β-hCG dynamics: PGT cycles vs. non-PGT cycles

**DOI:** 10.1007/s00404-025-08057-9

**Published:** 2025-05-31

**Authors:** Gonul Ozer, Meryem Hocaoglu, Sabri Berkem Okten, Semra Kahraman

**Affiliations:** 1 Department of Obstetrics and Gynecology, Uskudar University, Istanbul, Turkey; 2 Assisted Reproductive Technologies and Reproductive Genetics Centre, Istanbul Sisli Memorial Hospital, Istanbul, Turkey; 3 Obstetrics and Gynecology, Goztepe Prof. Dr. Suleyman Yalcin City Hospital Affiliated to Istanbul Medeniyet University, Istanbul, Turkey; 4 Obstetrics and Gynecology and Reproductive Medicine, Acibadem Kozyatagi Hospital, Istanbul, Turkey

**Keywords:** ß-Human chorionic gonadotropin, Frozen-thawed embryo transfer, Trophectoderm biopsy, Pre-implantation genetic test, Pregnancy

## Abstract

**Purpose:**

Pre-implantation genetic testing (PGT), which involves trophectoderm (TE) biopsy, is commonly used to detect genetic abnormalities in embryos. However, its impact on serum β-human chorionic gonadotropin (β-hCG) levels in early pregnancy remains a topic of debate. This study evaluated the effects of TE biopsy on β-hCG dynamics. Serum β-hCG levels on days 9 and 11 post-blastocyst transfer were compared between PGT and non-PGT cycles. Additionally, β-hCG thresholds were explored as potential prognostic markers for success in assisted reproductive technology (ART).

**Methods:**

This retrospective cohort study was conducted at the Memorial Şişli Hospital, İstanbul, Türkiye, between January 2012 and January 2021. The patients undergoing frozen-thawed single blastocyst transfer were divided into PGT (1698 cycles) and non-PGT (1830 cycles) groups. The serum β-hCG levels on days 9 and 11 after embryo transfer (ET) and the rate of β-hCG increase were compared.

**Results:**

In both groups, higher baseline β-hCG levels and rates of increase were correlated with live birth outcomes than with clinical or biochemical pregnancy loss (*p* < 0.001). PGT cycles showed lower baseline β-hCG levels across all pregnancy outcomes, but no significant difference in β-hCG increase rates (*p* > 0.05). After adjusting for confounding factors, PGT cycles were not found to be significantly associated with β-hCG levels.

**Conclusion:**

Serum β-hCG dynamics strongly predict live birth and clinical pregnancy. PGT did not significantly affect β-hCG levels after adjustment for confounders.

## What does this study add to the clinical work

**Table:** 

This study shows that the differences in β-hCG levels between the PGT and non-PGT groups are mainly influenced by confounding factors such as BMI, previous miscarriage, and blastocyst grading, rather than PGT itself. This emphasizes the need for controlling confounders in clinical studies and suggests that further research is needed to explore the mechanisms affecting early pregnancy outcomes after PGT.	

## Introduction

Women undergoing assisted reproductive technology (ART) treatment frequently face considerable anxiety and psychological stress during the period leading up to their initial β-human chorionic gonadotropin (β-hCG) test results. 81% report that this phase is highly or very stressful [21]. An accurate early predictor of pregnancy outcomes could reduce anxiety associated with the uncertainty of ART treatments. [12]. β-hCG is a hormone associated with pregnancy, produced by e-placental syncytiotrophoblasts, and is detectable in maternal serum as early as 8 days post-ovulation [11]. β-hCG levels approximately doubled every 48 h. Consequently, the increase in serum β-hCG concentration, measured at 2-day intervals, provides an essential predictor of pregnancy outcomes. This pattern has been observed in natural and in vitro (IVF) pregnancies [4, 33].

Pre-implantation genetic testing (PGT) is an ART procedure that involves obtaining a cellular sample from an embryo to determine the embryo’s ploidy status or to identify specific genetic abnormalities. This test helps to identify the most suitable embryo for transfer [3]. PGT was performed for monogenic disorders (PGT-M), structural rearrangements (PGT-SR), and aneuploidy screening (PGT-A) [34]. PGT can be performed using several methods, including polar body, blastomere, and trophectoderm (TE) biopsies. TE biopsy, the preferred method, offers the advantage of retrieving five–ten cells from a blastocyst for more reliable results. Unlike cleavage-stage biopsy, TE biopsy is believed not to adversely impact embryo development [14]. The inner cell mass of human blastocysts plays little or no role in β-hCG mRNA expression. In contrast, trophectoderm (TE) cells, which differentiate into syncytiotrophoblasts, are primarily responsible for the production and secretion of β-hCG [2, 24]. Therefore, the question arises as to whether the widely used PGT procedure involving TE biopsy affects the initial serum β-hCG level. Several studies with relatively small sample sizes have compared the initial serum β-hCG levels in PGT and non-PGT cycles [6, 20, 37]. Some clinical studies have reported that pregnant women who conceive after PGT cycles have lower initial serum β-hCG levels [18, 20]. In contrast, few studies have shown that TE biopsy does not affect the initial or subsequent β-hCG measurement [35, 37].

This study aimed to compare serum β-hCG levels and the rate of β-hCG increase on days 9 and 11 after blastocyst transfer as predictors of early pregnancy outcomes in both PGT and non-PGT cycles. The secondary objective was to determine cut-off values for baseline β-hCG levels and β-hCG increase rate in pregnancy outcomes.

## Materials and methods

### Ethical approval

This study was approved by the Institutional Review Board of the Istanbul Memorial Sisli Hospital, Istanbul, Türkiye (approval number 03.06.2023/003).

### Study design and population

This retrospective cohort study was conducted at the Assisted Reproductive Technologies and Reproductive Genetics Center at Memorial Sisli Hospital in Istanbul, covering the period from January 2012 to January 2021. Patients with positive serum β-hCG results (≥ 20 IU/L) after frozen–thawed single blastocyst transfer were divided into two groups: PGT group (1698 cycles) and non-PGT group (1830 cycles). Serum β-hCG levels on days 9 and 11 after embryo transfer (ET) and the rate of β-hCG increase were compared between the PGT and non-PGT cycles. Cycles without ET and patients with incomplete data were excluded from the analysis. The demographic and clinical characteristics of the patients were recorded (Table 1).

**Table 1:** Baseline characteristics of the study participants

Characteristics	PGT group (*n* = 1698)	Non PGT group (*n* = 1830)	*p*-value
Maternal age, years	34.54 ± 4.98	30.47 ± 4.10	< 0.001*
Paternal age, years	37.38 ± 5.95	34.10 ± 4.92	< 0.001*
BMI, kg/m^2^	24.53 ± 4.38	24.97 ± 4.92	0.006*
Duration of infertility, months	58.30 ± 50.84	60.23 ± 43.52	< 0.001*
Anti-Müllerian hormone, ng/mL	2.98 ± 2.58	4.37 ± 3.56	< 0.001*
Number of retrieved oocytes	13.30 ± 8.84	17.31 ± 9.64	< 0.001*
Number of metaphase II oocytes	11.46 ± 7.45	14.46 ± 9.64	< 0.001*
Blastocyst grading n (%)			< 0.001*
Top	985 (%58)	1277(%69)	
Good	625 (%37)	515(%28)	
Moderate	68 (%25)	20(%10)	
Poor	18(%10)	10(%5)	
Endometrial preparation protocols, n (%)			< 0.001*
mNC-FET	1039(%61)	1017(%56,6)	
ERT-FET	659 (%39)	813(%44)	
Blastocysts transfer, n (%)			< 0.001*
Day 5	1593(%93)	1805(%98)	
Day 6	105(%6)	25 (%13)	
Endometrial thickness on transfer day, mm	10.19 ± 3.31	10.35 ± 1.82	< 0.001*

*PGT* preimplantation genetic testing; *BMI* body mass index; m*NC-FET* modified natural cycle frozen embryo transfer; *ERT-FET* estrogen replacement therapy frozen embryo transfer; *β-hCG* human chorionic gonadotropin; *ET* embryo transfer

Data are presented as either mean ± standard deviation or number (%)

Chi-square and Mann–Whitney *U* tests were used for categorical and continuous variables, respectively

Phi and Cramer’s V test was used for categorical variables in effect size measurement

*p < 0.05 was significant

### Treatment protocols and clinical procedures

The stimulation protocols are detailed in our previous study [13]. Ovarian stimulation was performed using either a gonadotropin-releasing hormone (GnRH) analog protocol or a GnRH antagonist protocol. For this purpose, the recombinant follicle-stimulating hormone (FSH) (GONAL-f; Merck Serono, Switzerland), a combination of FSH and recombinant luteinizing hormone (LH) (Luveris; Merck Serono, Switzerland), or human menopausal gonadotropin (hMG; Ferring, Switzerland) was administered. Oocyte retrieval was performed via transvaginal ultrasound 36 h after the administration of 250 µg of recombinant human chorionic gonadotropin (rHCG; Ovitrelle; Merck Serono, Switzerland) or a GnRH analog (Lucrin; Abbott Laboratories, USA). Intracytoplasmic sperm injection (ICSI) was used as the fertilization method [36]. The embryos were evaluated 114–120 h after ICSI using the Gardner scoring system. This assessment classifies embryos into three categories: top quality (TQ), good quality (GQ), and moderate/poor quality (PQ) blastocysts [10]. TQ-GQ blastocysts (graded at a minimum of 3 BB) were frozen after TB. Vitrification and thawing were conducted using Kitazato vitrification/thawing media (Kitazato, Japan) and Cryotop® as the carrier, following the manufacturer’s protocol. Embryo thawing and transfer were scheduled 6 days after the LH surge. Following the thawing process, embryos were initially assessed for viability 30 min after thawing and subsequently re-evaluated 2 h later for various indicators, including hatching, re-expansion, necrotic foci, and extensive cytoplasmic granulation. These indicators are predictive of implantation rates [7]. Blastocysts that demonstrated at least 80% re-expansion and confirmed viability were transferred to the same day. Frozen-thawed embryo transfers were conducted following endometrial preparation via either a modified natural cycle (mNC-FET) or estrogen replacement therapy (ERT-FET), as previously outlined in our study [27]. Micronized vaginal progesterone gel was administered for luteal phase support during the FET cycles. Luteal support was administered until a positive pregnancy test result was obtained and continued until the 10 th week of gestation.

### Trophectoderm biopsy

TB for PGT using next-generation sequencing (NGS) was performed on blastocysts exhibiting at least 3BB expansion (according to Gardner's classification) on day 5 or 6. Zona opening was carried out on day 3 or 4 to facilitate the procedure. Biopsy was performed in pre-warmed 10% HSA-supplemented HEPES-buffered drops (Global®, CooperSurgical, USA) at 37 °C under paraffin oil. A holding pipette stabilized the blastocyst opposite the herniated cells, whereas a biopsy pipette and diode laser (Saturn 3, RI Cooper Surgery, USA) excised 5–8 trophectoderm cells. The cells were then washed in PBS, placed into 0.2 ml PCR tubes, and stored at −20 °C until they were ready for PGT analysis.

### Pregnancy outcomes and common terminology

Initial serum β-HCG levels were measured on day 9 post-blastocyst transfer, and values ≥ 20 IU/L were considered positive. The subsequent tests were performed on day 11. Biochemical pregnancy was indicated by a positive β-HCG result without a visible gestational sac. By week 7, clinical pregnancy was identified via detected fetal heartbeats, and ongoing pregnancy by a viable fetus at week 12. Live births were recorded when at least one baby was born. Clinical pregnancy loss was defined as the loss of pregnancy after the formation of a gestational sac but before the 20th week, whereas biochemical pregnancy loss occurred before the detection of a sac. Biochemical pregnancy loss (BPL) refers to the loss of pregnancy prior to the detection of an intrauterine gestational sac.

### Power analysis and sample size

Power analysis was performed using G*Power software (version 3.1.9.7) [9] to determine the sample size required to detect a 20% effect size for the goodness-of-fit tests, with a target power of 95% and a significance level of *α* = 0.05. The analysis indicated that 1302 individuals were necessary for the study.

### Statistical analysis

Statistical analyses were conducted using SPSS software (version 22.0; IBM, NY, USA). Continuous numerical attributes were expressed as mean (standard deviation [SD]); the categorical attributes were reported as number and percentage. The distribution of data was calculated using the Kolmogorov–Smirnov test for continuous attributes. Categorical attributes were compared using the chi-square test (with Bonferroni adjustments). The Mann–Whitney U test and Kruskal–Wallis test were used to detect the relationship between continuous and categorical variables. To evaluate the independent factors influencing initial serum β-hCG levels, univariate and multivariate linear regression analyses were performed (Tables 3, 4). This model included variables that were found to be statistically significant in univariate analyses and adjusted for potential confounders, including maternal age, BMI, duration of infertility, endometrial thickness on the transfer day, history of prior miscarriage, and blastocyst grading. This approach allowed for a more accurate assessment of the association between PGT and serum β-hCG levels by accounting for relevant covariates. In addition, receiver operating characteristic (ROC) curve analysis was performed to predict pregnancy outcomes using the initial β-hCG level and rate of increase. Statistical significance was set at *p* < 0.05.

## Results

### Baseline characteristics

**Fig. 1:**
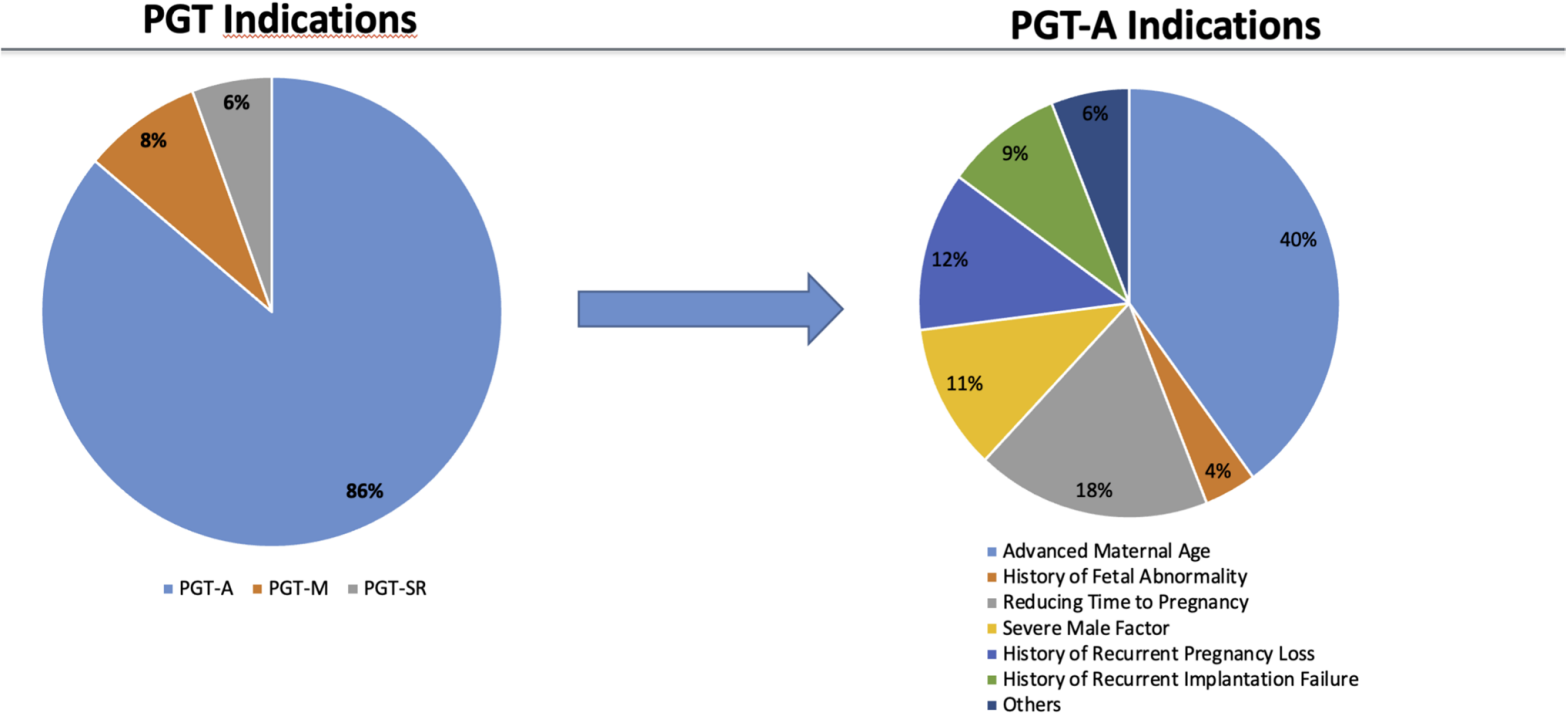
Preimplantation Genetic Test Indications

The main reason for conducting PGT was PGT-A, which comprised 86% of the cases. This was PGT-M at 8% and PGT-SR at 6%. Among the indications for PGT-A, the leading reason was advanced maternal age (40%), followed by the desire to reduce the time to pregnancy (18%) and a history of recurrent pregnancy losses (12%) (Fig. 1). Table 1 presents the baseline characteristics of the study participants. Maternal age, paternal age, and good-quality blastocysts were significantly higher in the PGT group than in the non-PGT group (*p* < 0.001). Conversely, the PGT group showed significantly lower duration of infertility (*p* < 0.001), AMH levels (*p* < 0.001), number of retrieved oocytes (*p* < 0.001), and metaphase II oocytes (*p* < 0.001), compared to the non-PGT group (Table 1).

### Comparison of the initial serum β-hCG levels and the rate of β-hCG increase based on pregnancy outcomes

Table 2 compares β-hCG level dynamics and pregnancy outcomes between the PGT and non-PGT groups. While the rate of increase in β-hCG levels did not show significant differences between the groups across outcomes, the initial β-hCG levels were consistently lower in the PGT group. Significant differences were observed in the initial β-hCG levels for biochemical pregnancy loss (*p* = 0.041), clinical pregnancy loss (*p* = 0.040), clinical pregnancy (*p* = 0.006), ongoing pregnancy (*p* = 0.002), and live birth (*p* = 0.004), favoring higher levels in the non-PGT group.

**Table 2:** Comparison of the initial level and rate of increase in serum β-hCG according to pregnancy outcomes between the PGT and non-PGT groups

	Increase rate of β-hCG levels (fold)	PGT group (*n* = 1698)	Non-PGT group (*n* = 1830)	P value	Initial β-hCG levels (IU/L)	PGT group (*n* = 1698)	Non-PGT group (*n* = 1830)	P value
Biochemical pregnancy loss	Mean ± SD	1.39 ± 1.07	1.60 ± 1.83	0.125	Mean ± SD	66.81 ± 55.92	72.25 ± 50.82	0.041*
Clinical pregnancy loss	Mean ± SD	2.60 ± 1.16	2.59 ± 0.99	0.347	Mean ± SD	137.64 ± 89.08	155.71 ± 98.32	0.040*
Clinical pregnancy	Mean ± SD	2.72 ± 0.82	2.68 ± 0.78	0.354	Mean ± SD	177.34 ± 98.86	184.66 ± 98.04	0.006*
Ongoing pregnancy	Mean ± SD	2.74 ± 0.77	2.64 ± 0.52	0.988	Mean ± SD	182.81 ± 98.50	191.05 ± 96.47	0.002*
Live birth	Mean ± SD	2.73 ± 0.75	2.71 ± 0.73	0.300	Mean ± SD	183.86 ± 99.56	191.52 ± 97.11	0.004*

Initial β-HCG levels were measured on day 9 post-blastocyst transfer

Values are presented as mean ± standard deviation. An asterisk (*) indicates statistical significance at p <0.05

### Impact of PGT on serum β-hCG levels

Tables 3 and 4 presents the results of the linear regression analyses evaluating the effect of PGT and other variables on initial serum β-hCG levels. Model 1 (univariable analysis) demonstrated a statistically significant association between PGT and lower β-hCG levels (standardized coefficient = 0.039, *p* = 0.029). However, in Model 2 (multivariate analysis), which was adjusted for maternal age, BMI, duration of infertility, endometrial thickness on the day of embryo transfer, prior spontaneous miscarriage, and blastocyst grading, the association between PGT and β-hCG levels was no longer statistically significant (standardized coefficient = 0.031, *p* = 0.069). In contrast, BMI (*β* = −0.182, *p* < 0.001), history of previous miscarriage (*β* = −0.195, *p* < 0.001), and blastocyst quality (*β* = 0.133, *p* < 0.001) remained independently associated with β-hCG levels in Model 2.

**Table 3:** Univariate linear regression analysis (Model 1) of factors associated with initial serum β-hCG levels

Variable	Unstandardized Coefficient	Standardized Coefficient	*t*-value	*p*-value
PGT vs. non-PGT	−7.942	0.039	2.181	0.029*
Maternal age	0.202	0.022	0.711	0.476
BMI	−4.094	−0.175	−10.731	< 0.001*
Duration of infertility	−0.032	−0.018	−1.010	0.312
Endometrial thickness	−0.060	−0.021	−1.471	0.145
Previous spontaneous miscarriage	−27.004	−0.205	−11.721	< 0.001*
Day of blastocyst transfer	−16.439	−0.037	−2.037	0.042*
Blastocyst grading	9.585	0.141	8.540	< 0.001*

Asterisk (*) indicates statistical significance, defined as p < 0.05

**Table 4:** Multivariate linear regression analysis (Model 2) of factors independently associated with initial serum β-hCG levels

Variable	Unstandardized Coefficient	Standardized Coefficient	*t*-value	*p*-value
PGT vs. non-PGT	6.240	0.031	1.820	0.069
Maternal age	0.202	0.010	0.541	0.588
BMI	−4.094	−0.182	−11.028	< 0.001*
Duration of infertility	−0.032	−0.015	−0.870	0.384
Endometrial thickness	−0.060	−0.026	−1.604	0.109
Previous spontaneous miscarriage	−27.004	−0.195	−11.997	< 0.001*
Day of blastocyst transfer	−16.439	−0.031	−1.885	0.059
Blastocyst grading	9.585	0.133	8.131	< 0.001*

Initial β-HCG levels were measured on day 9 post-blastocyst transfer

Asterisk (*) indicates statistical significance, defined as p < 0.05

### The ROC curve analysis of the initial serum β-hCG levels and its rate of increase

**Fig. 2:**
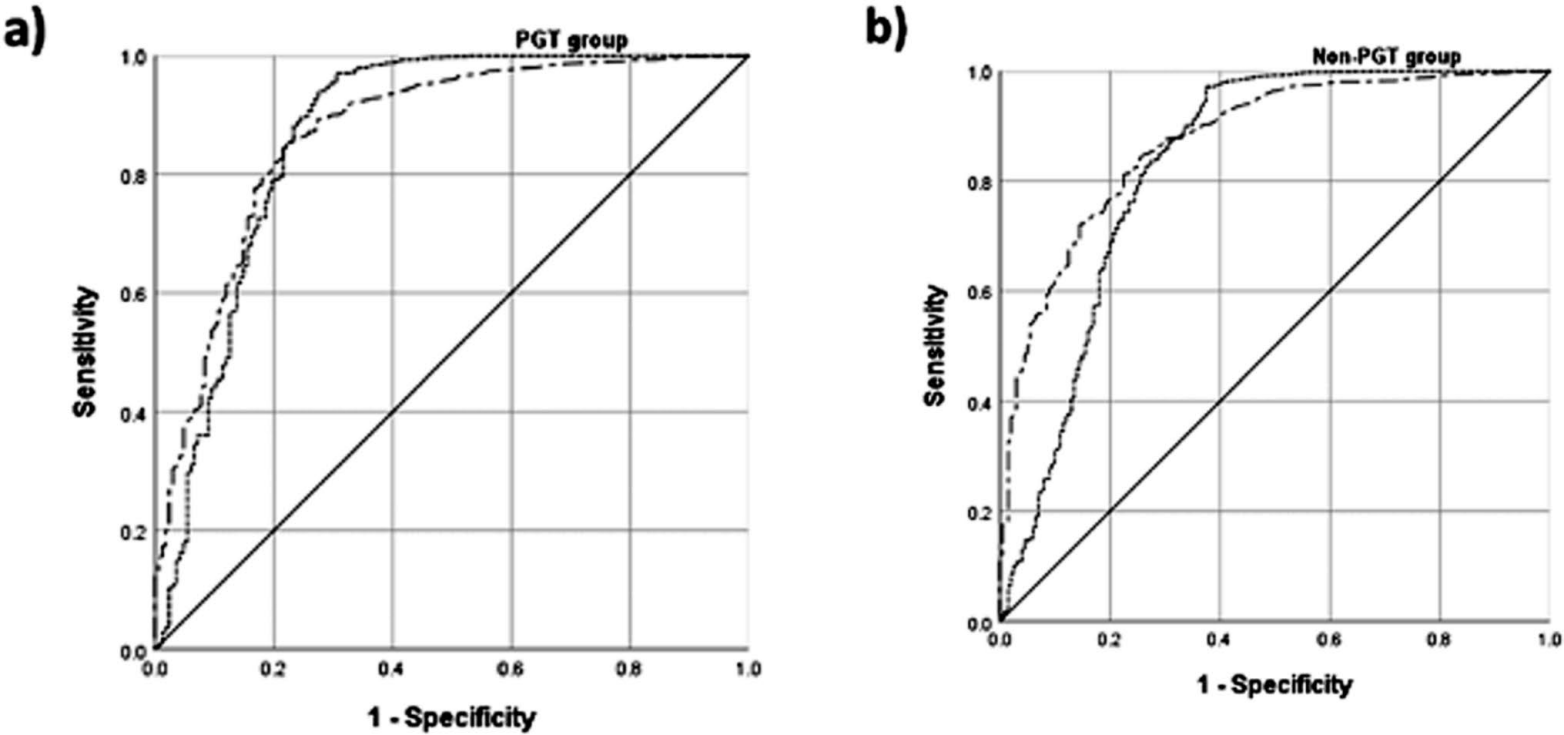
Prediction of clinical pregnancy based on baseline serum β-hCG levels and their rate of increase using ROC curve analysis: **a** PGT group; **b** non-PGT group

ROC analysis was used to evaluate the predictive performance of the initial β-hCG levels and fold increases (Table 5). The PGT group showed higher sensitivity and PPV for all outcomes. In contrast, the non-PGT group demonstrated greater specificity and NPV, especially for ongoing pregnancies and live births. The AUC values were higher in the PGT group overall (*p* < 0.001). Notably, the β-hCG increase rate had limited predictive value for ongoing pregnancy in the PGT group (AUC = 0.535). Figure 2 presents the clinical pregnancy prediction based on baseline serum β-hCG levels and its rate of increase using ROC curve analysis: part (a) represents the PGT group and part (b) the non-PGT group.

**Table 5:** Prediction of live birth, clinical pregnancy, and ongoing pregnancy by the initial level and rate of increase in serum β-hCG levels between patients after PGT and non-PGT

Variables	Initial serum β-hCG levels, PGT group (*n* = 1698)			Initial serum β-hCG levels, Non PGT group (*n* = 1830)		
	Clinical pregnancy	Ongoing pregnancy	Live birth	Clinical pregnancy	Ongoing pregnancy	Live birth
Threshold value (IU/L)	71.5	101.50	122.5	100.5	121.5	130.5
Sensitivity (%)	89.3	80.0	70.1	81.5	75.5	71.7
Specificity (%)	72.5	60.6	60.1	76.0	61.0	61.6
PPV (%)	96.7	82.2	84.7	96.5	63.1	82.2
NPV	42.5	29.1	38.9	33.5	72.8	46.9
AUC	0.870	0.761	0,724	70.1	0.741	0,726
*p*-value	< 0.001	< 0.001	< 0.001	< 0.001	< 0.001	< 0.001

	Increase rate of β-hCGlevels (fold) PGT group (*n* = 1698)			Increase rate of β-hCGlevels (fold) Non PGT group (*n* = 1830)		
Threshold value (Fold)	1.98	2.47	2.33	2.11	2.5	2.31
Sensitivity (%)	90.6	58.3	73.5	83.6	52.0	73.6
Specificity (%)	74.3	45.1	59.9	72.5	47.9	52.9
PPV (%)	97.0	70.0	85.4	96.1	65.0	79.4
NPV (%)	46.3	98.0	41.4	35.1	98.6	44.9
AUC	0.869	0.535	0.705	0.831	0.542	0.662
*p*-value	< 0.001	< 0.001	< 0.001	< 0.001	< 0.001	< 0.001

Initial β-HCG levels were measured on day 9 post-blastocyst transfer

## Discussion

This study found that initial β-hCG levels were lower in PGT cycles than in non-PGT cycles across all pregnancy outcomes. However, after adjusting for confounding variables, including BMI, prior miscarriage, and blastocyst grade, the association was no longer statistically significant. These findings suggest that PGT itself may not directly affect β-hCG secretion and that the observed differences are likely due to patient or embryo characteristics.

Some previous studies reported no significant difference in β-hCG levels between PGT and non-PGT cycles, although the measurement timing varied. For instance, Wu et al. assessed β-hCG levels 14 days after embryo transfer and found no significant differences between the PGT and non-PGT groups. In their study, the clinical pregnancy cutoff was 482 IU/mL in the PGT group and 302 IU/mL in the non-PGT group (*p* = 0.989). Although the difference was not statistically significant, the cutoff value for live births was lower in the PGT group (1345 IU/mL) than in the non-PGT group (1621 IU/mL). This temporal variation may explain the higher cutoff values observed in their study than in ours [37]. Li et al. evaluated β-hCG levels 12 days after blastocyst transfer and reported no significant difference between the PGT and non-PGT groups (769 mIU/mL vs. 753 mIU/mL, *p* = 0.631). However, a notable difference was observed in subgroups with lower trophectoderm (TE) grades. Among blastocysts with TE grades B and C, the non-PGT group showed higher β-hCG levels than the PGT group (690 vs. 649 mIU/mL, *p* = 0.001 and 586 vs. 509 mIU/mL, *p* < 0.001, respectively). These results indicate that trophoblast biopsy may adversely affect β-hCG levels in blastocysts with TE grades B or C, but not in those with higher TE grades [18]. Similarly, Özdamar et al. reported that the β-hCG test results 15 days after embryo transfer were lower in the PGT group than in the non-PGT group. However, this difference was not statistically significant (PGT group = 1009.0 IU/L, non-PGT group = 1330.0 IU/L, *p* = 0.336) [28].

Some studies have reported findings consistent with ours. For instance, Lu et al. compared clinical pregnancy rates after the transfer of single embryos with and without TB. Their results indicated that the PGT group had significantly lower mean β-hCG levels (703.1 ± 569.6 vs. 809.2 ± 582.0 mIU/mL; *p* = 0.004) [20]. These mixed results emphasize the importance of standardized timing and adjustment for confounders when interpreting β-hCG trends. Regression analyses revealed that while PGT was associated with lower β-hCG levels in univariate analysis, the effect was attenuated after adjustment for confounders in the multivariate model. Notably, BMI, blastocyst quality, and previous miscarriages remained significant predictors. This underscores the multifactorial nature of β-hCG dynamics and supports adjusting for patient- and embryo-related factors in predictive modeling.

Multiple regression analysis revealed a significant negative relationship between BMI and serum β-hCG levels, with each unit increase in BMI associated with a 4.094 unit decrease in β-hCG levels (*p* < 0.001). This finding is in line with previous studies that have demonstrated a negative correlation between BMI and β-hCG levels [1, 8, 22, 32]. In addition, it is well established that fertility is diminished in overweight and obese women, who also exhibit a higher risk of miscarriage, further emphasizing the detrimental impact of elevated BMI on reproductive outcomes [29]. Several explanations have been proposed for the lower β-hCG concentrations observed in obese women. One hypothesis is that obese women may have a larger extracellular volume, which leads to a dilution effect and, consequently, lower β-hCG values [23]. Another potential explanation involves the sequestration of β-hCG by macrophages within adipose tissue, which may reduce circulating β-hCG levels. Furthermore, obesity is associated with impaired trophoblast differentiation and reduced angiogenesis, which could further contribute to lower β-hCG levels in women with a higher BMI [30].

Blastocyst morphology was also identified as a significant factor influencing β-hCG levels in our multiple regression analysis. Consistent with previous studies, we observed that higher-quality blastocysts were associated with higher β-hCG levels [16, 18, 25, 35] Li et al. analyzed 7847 single blastocyst transfers and found that poor blastocyst morphology, particularly low trophectoderm grade, was associated with lower β-hCG values. Differentiated syncytiotrophoblasts in the trophectoderm secrete β-hCG, and β-hCG concentration in the blood is likely related to trophectoderm quality. Furthermore, our study suggests that a history of miscarriage has a statistically significant, potentially negative impact on β-hCG levels. Several studies have indicated that low initial hCG levels are associated with an increased risk of miscarriage [15, 25, 26, 31]. β-hCG levels may be influenced by hormonal imbalances or endometrial conditions resulting from previous miscarriages [5]. Furthermore, the recurrent miscarriages are often associated with other complications, such as immune reactions that affect implantation and chromosomal abnormalities that may prevent embryonic development [17, 19].

## Limitations

This study had some limitations. First, its retrospective design introduces the potential for selection bias and limits causal inferences. Although major confounders such as BMI, blastocyst quality, and prior miscarriage were adjusted for, residual confounding from unmeasured variables, such as subtle differences in embryo metabolism, uterine receptivity, or biopsy timing, may still exist. Additionally, while initial β-hCG levels showed a strong predictive value, the rate of β-hCG increase demonstrated limited accuracy in forecasting ongoing pregnancy in the PGT group. This highlights the need for cautious interpretation of dynamic β-hCG trends. Future prospective multicenter studies are necessary to validate these findings and further elucidate the biological impact of trophectoderm biopsy on early implantation physiology.

## Conclusion

This study demonstrated that although initial β-hCG levels were consistently lower in PGT cycles, this difference was no longer statistically significant after adjusting for key confounding variables, such as BMI, blastocyst morphology, and prior miscarriage. These findings suggest that the observed variations in β-hCG dynamics are more likely due to patient- and embryo-related characteristics rather than the direct effect of trophectoderm biopsy itself. Therefore, clinicians should interpret early β-hCG measurements within a multifactorial context, especially when managing expectations after PGT cycles. Future prospective and multicenter studies are essential to validate these findings and further investigate the underlying biological mechanisms that may influence early implantation and hormonal profiles following embryo biopsy.

## Data Availability

No datasets were generated or analysed during the current study.

## Abbreviations

PGT: Pre-implantation genetic testing
TE: Trophectoderm
TB: Trophectoderm biopsy
β-hCG: β-Human chorionic gonadotropin
ART: Assisted reproductive technology
ET: Embryo transfer
FET: Frozen-thawed embryo transfers
BMI: Body mass index
BPL: Biochemical pregnancy loss
CPL: Clinical pregnancy loss
ROC: Receiver operating characteristic curve analysis
AUC: Area under receiver operating characteristic curve
PPV: Positive predictive value
NPV: Negative predictive value
OR: Odds ratio
mNC-FET: Modified natural cycle frozen embryo transfer
ERT-FET: Estrogen replacement therapy frozen embryo transfer
GnRH: Gonadotropin-releasing hormone
FSH: Follicle-stimulating hormone
ICSI: Intracytoplasmic sperm injection
TQ: Top quality
GQ: Good quality
PQ: Moderate/poor quality
